# C3a-C3aR signaling promotes breast cancer lung metastasis via modulating carcinoma associated fibroblasts

**DOI:** 10.1186/s13046-019-1515-2

**Published:** 2020-01-13

**Authors:** Chi Shu, Haoran Zha, Haixia Long, Xinxin Wang, Fei Yang, Jianbao Gao, Chunyan Hu, Li Zhou, Bo Guo, Bo Zhu

**Affiliations:** 1 Institute of Cancer, Xinqiao Hospital, Third Military Medical University, Chongqing, 400037 People’s Republic of China; 2 Chongqing Key Laboratory of Immunotherapy, Chongqing, 400037 People’s Republic of China; 3 Department of Oncology, The General Hospital of the PLA Rocket Force, Beijing, 100088 People’s Republic of China; 4 Maternal & Child Health Research Institute, Baoan Women’s and Children’s Hospital, Jinan University, Shenzhen, 518101 People’s Republic of China

**Keywords:** C3a, C3a receptor, Complement, Cancer-associated fibroblast, Metastasis, Breast cancer

## Abstract

**Background:**

Mounting evidence suggests that complement components promote tumor progression via modulating immune suppression, angiogenesis, or tumor cell proliferation. However, the role of C3a-C3aR signaling in regulating lung metastasis of breast cancer remains unknown.

**Methods:**

We performed various ex-vivo and in-vivo assays. Genetic and pharmacological C3aR blockade models were applied to investigate the role of C3a-C3aR in metastasis of breast cancer.

**Results:**

C3a-C3aR signaling in CAFs facilitates the metastasis of breast cancer. Mechanically, C3a-C3aR signaling augments pro-metastatic cytokine secretion and extracellular matrix components expression of CAFs via the activation of PI3K-AKT signaling. Genetic or pharmacological blockade of C3aR signaling effectively inhibited lung metastasis of breast cancer in mouse models.

**Conclusions:**

C3a-C3aR signaling in CAFs facilitates the metastasis of breast cancer. Targeting C3aR signaling is a potential anti-metastasis strategy for breast cancer therapy.

## Background

The complement system is an important part of innate immunity, which consists of a series of serine proteases encoded by the same ancestral gene as the coagulation protein [1]. In recent years, the role of the complement system has shifted from a blood- based antimicrobial infection to a wide range of immune regulation and tissue homeostasis regulation [2–4]. In addition to participating in innate immunity, many complement proteins facilitate cross-talk between the immune cells and tumor cells in the tumor microenvironment (TME) [5–7]. Complement C3 is a central component in complement activation [1, 8], activation of C3 results in the generation of C3a, which is a prominent tumor-promoting factor in TME [9, 10]. Numerous studies have shown that myeloid cells (including monocytes, macrophages, DCs) and T cells express C3aR [7, 11]. In addition, activated astrocytes, endothelial cells, epithelial cells [12], smooth muscle cells [2], renal tubular epithelial cells [13] are also regulated by the C3a-C3aR axis. Previously, we and others demonstrated that C3aR signaling promotes tumor growth by promoting immune inhibition [6, 10, 14]. However, the role of C3a-C3aR signaling in breast cancer metastasis remains to be explored. Published studies suggested that C3a-C3aR signaling contributes to the formation of pulmonary fibrosis [15] and renal fibrosis [16], characterized by activation of fibroblasts, which reveal the potential of C3a-C3aR axis in promoting metastasis via regulating fibroblasts in TME.

A growing body of evidence suggests that tumor metastasis is not only dependent on tumor cells themselves, but is also regulated by the tumor microenvironment (TME) [17]. Carcinoma associated fibroblasts (CAFs) are the largest populations of tumor cells which accumulate in TME [18, 19] (e.g. breast cancer [20], hepatocellular carcinoma [21]) and promote cancer metastasis through multiple pathways [22, 23]. Manipulating the function of CAFs is a promising strategy to treat cancer [24]. However, whether and how C3a-C3aR signaling is involved in the regulation of CAFs remain largely unknown.

In the current study, we demonstrated that C3a promotes tumor cell metastasis by modulating CAFs. C3a binds to its cognate receptor C3aR to activate PI3K/AKT signaling, which resulted in CAFs activation. Moreover, in human invasive breast cancers, C3 expression is positively correlated with expression of CAFs activation markers and functional effectors. Genetic or pharmacological blockade of C3aR signaling effectively inhibits lung metastasis of breast cancer. Our data demonstrated that targeting C3aR might be an effective strategy in tumor metastasis control.

## Material and methods

### Cell lines and cell cultures

The mouse breast cancer cell lines 4 T1 and EO771 used in this study were obtained from the American Tissue Culture Collection (ATCC) and CH3 BioSystems respectively. Cells were routinely cultured in the recommended high-glucose DMEM medium (Hyclone) supplemented with 10% fetal bovine serum (Hyclone), L-glutamine (2 mM), penicillin (100 mg/mL), and streptomycin (100 mg/L). Primary CAFs were isolated from 4 T1 breast tumor tissues of BalB/c mice or C3a receptor-deficient (C3aR^−/−^) mice by FACS. All cultures were incubated at 37 °C in a humidified atmosphere of 5% CO_2._

### Mice

Six- to 8-week-old female Wild-type BalB/c mice were purchased from the Chinese Academy of Medical Sciences (Beijing, China). C3aR^−/−^ mice with a BalB/c background were kindly provided by Dr. Zhou Hong (Department of Immunology, Nanjing Medical University). The mice were kept under specific pathogen-free conditions at the Animal Center of Third Military Medical University. Six- to 8-week-old female nude mice were purchased from the Animal Institute of the Academy of Medical Science (Beijing, China). The MMTV-PyMT mice were kindly provided by Dr. Liu Xiaolong (Institute of Biochemistry and Cell Biology, Shanghai Institutes for Biological Sciences, Chinese Academy of Sciences). For sacrificing the mice, they were kept in a chamber with isoflurane (concentration: 3–4%) for 2–3 min. After the induction of anesthesia, mice were immediately sacrificed by cervical dislocation. The animal studies have been conducted in accordance with the guidelines of the Institutional Animal Care and Use Committee (IACUC) of the Third Military Medical University [10].

### The orthotopic transplanted tumorigenicity

The 4 T1 cells were routinely cultured with DMEM containing 10% fetal bovine serum until they reached 70–80% confluency. Thereafter, 6 to 8-week-old female C3aR^−/−^ (Balb/c background) or Balb/c mice were orthotopically injected with 1 × 10^5^ 4 T1 cells in 100 μl phosphate-buffered saline per mice in the third mammary fat pad at day 0. Tumor sizes were monitored two or three times per week from day 7,and tumor volume was calculated as follows: V = (length × width^2^) × 0.5 [25]. The mice were anesthetized and sacrificed 28 days after tumor inoculation. Primary tumors were dissected from the body and weighed, and tumor weight and volume were analyzed. There were six to eight mice in each group.

### Lung metastasis assays

For the orthotopic model of spontaneous metastasis, 4 T1 cells were inoculated into the third mammary fat pad of WT or C3aR^−/−^ mice on day 0, and tumor sizes were monitored three times a week. The mice were anesthetized and sacrificed on day 28 post tumor injection. Indian ink was injected into the lungs of sacrificed mice with a syringe through the trachea until the lungs were completely filled with ink. The lungs were then removed immediately and immersed in Fekete’s solution for several minutes to show the pulmonary nodules; white dots on the black lungs were observed and counted for tumor metastasis. The MMTV-PyMT mice were sacrificed at 16 weeks of age, and tumor nodules in the lungs were counted after India ink injection as indicated above.

For the co-implantation assay, a total of 5 × 10^4^ 4 T1 cells alone or mixed with 2.5 × 10^5^ sorted CAFs from either WT or C3aR^−/−^ mice were co-injected into the third mammary fat pad of 6 week old female Balb/c nude mice. Tumor sizes were measured twice or three times a week and tumor volumes were calculated 28 days later, mice were anaesthetized and sacrificed. Filling the lungs with ink so that metastatic nodules were visible, the number of metastases was compared among three groups, each of which used 5–7 mice.

### C3aR antagonism treatment

A selective antagonist of C3aR (C3aRA, SB290157, Cayman) was used for C3aR blockade. 4 T1 tumor-bearing mice were randomly assigned to control or experimental groups on day 1 post tumor inoculation and were intraperitoneally injected with C3aRA (10 mg/kg body weight) or PBS twice weekly, the mice were euthanized and sacrificed 28 days after tumor injection and the lung nodules were calculated and analyzed.

The MMTV-PyMT mice were treated with C3aRA(10 mg/Kg bodyweight) in 200 ul or PBS as early as 4-weeks of age, and they were sacrificed at 16 weeks of age. Lung metastases were identified and compared between the C3aRA treatment and control groups.

### Western blot

Protein was extracted from the cells or tumor tissues with RIPA buffer, dissolved with SDS–polyacrylamide gels and transferred to PVDF membranes (Millipore, 0.45 μm). The membranes were then blocked with 5% BSA at 37 °C for 1 h and incubated with primary antibodies at 4 °C overnight. Primary antibodies against a-SMA (clone: E184, Abcam,1:1000 diluted), p-AKT-Ser473 (clone: D9E, CST, 1:1000 diluted), AKT (CST, 1:1000), GAPDH (Beyotime, AF0006, 1:1000 diluted), Vimentin (Beyotime, AF0318, 1:1000 diluted), and E-Cadherin (Beyotime, AF0138,1:1000 diluted),α-Tubulin Rabbit Polyclonal Antibody (Beyotime, AF0001, 1:1000 diluted), were used. After washing with 0.1% TBST several times, the membranes were incubated with goat anti-rabbit secondary antibodies (Beyotime, 1:5000) or goat anti-mouse secondary antibodies (Beyotime, 1:5000) for 1 h at room temperature. The protein expression levels were visualized by the enhanced chemo-luminescence assay (ECL, Beyotime). Images were captured by using FluorChem HD2 system.

### ELISA assay

For in vitro stimulation of fibroblasts assay, sorted fibroblasts were seeded in 24-well plates in DMEM containing 10% FBS and 100 units/ml penicillin/streptomycin until 80% confluency. After washing with PBS, the cells were cultured in fresh serum-free media or stimulated with different doses of recombinant mouse C3a (rmC3a) (R&D). Supernatants were collected 48 h later, and filter sterilized using a 0.22 μm filter. The TGFβ1-ELISA kit (Dakewe, 1,217,102) was used for TGF-β1 detection. All experiments were performed according to the manufacturer’s instructions.

### Quantitative PCR and RNA sequencing

The RNA extraction of C3aR^+/+^ or C3aR^−/−^ CAFs were performed as previously described in [25]. In brief, 500 ng of RNA was reverse-transcribed using the PrimeScript™ RT Master Mix kit (Takakra). Real-time PCR was conducted with SYBR Premix Ex Taq™ II (Takara) to quantify the relative expression of mRNA. All primer sequences for real-time qPCR are shown in (Additional file 1: Table S1). Relative changes were quantified using the 2 − ΔΔCT method [26].

For RNA sequencing, after RNA collection from four WT tumors and four C3aR^−/−^ tumor tissues, sequencing libraries were generated using NEBNext® UltraTM RNA Library Prep Kit for Illumina® (NEB, USA). The library preparations were sequenced on an Illumina Hiseq platform and was performed by Novogene (Beijing, China). The DESeq2 R package was used for differential expression analysis of two groups. The clusterProfiler R package was used to conduct Gene Ontology (GO) enrichment analysis on differentially expressed genes to correct gene length deviation. The significance of the differential gene expression was as setting the *p* value threshold at 0.05.

### Analysis of the 2012 Cancer genome atlas (TCGA) data set

The mRNA profiles for tumor samples of 526 invasive breast cancer patients were downloaded in February 2019 from the cBioPortal for cancer genomics (www.cbioportal.com) [27–29]. Spearman’s correlation analyses were conducted to determine the correlation between C3 expression and expression of CAF markers and functional cytokine transcript levels. *P*-values < 0.05 were considered statistically significant.

### Transwell and wound-healing assays

Transwell assays were performed in 24-well inserts (Falcon 8.0-μm pore size, Corning) for migration or invasion assays. 4 T1 or EO771 cells were serum starved overnight. Then, 2 × 10^4^ tumor cells were plated in transwell inserts or matrigel-coated inserts as previously described [30], followed by co-culturing with 1 × 10^5^ CAFs isolated from WT mice or C3aR^−/−^ mice for 24 h. Cells in the upper part of the transwells were removed with a cotton swab, and the migrated cells were fixed in 4% paraformaldehyde and stained with 0.5% crystal violet. The membrane was observed under the microscope and photographed to calculate the total number of cells. Each experiment was repeated at least three times independently.

For the wound-healing assay, 2 × 10^5^/wells of 4 T1 were seeded in six-well plates. A pipette tip was used to draw a gap on the plates. The sorted WT CAFs or C3aR^−/−^ CAFs were plated in the upper inserts. The migration of 4 T1 to the blank area was observed under the microscope and imaged at a specific time point.

### Flow cytometry assays and CAFs isolation

The mammary tumors were dissected from the mice at the indicated time points. The tissues were cut into pieces and digested with collagen IV (1 mg/ml, sigma) and Dispase II (1 mg/ml, Sigma) and they were shaking for 1 h at 37 °C. The tissue/media mixture was strained using a 70 μm cell strainer for single cell suspension preparation. The cells were added anti-mouse CD16/CD32 (Clone 93, Biolegend) and incubated on ice for 10 min. Thereafter they were labeled with anti-mouse PDGFRα (Clone APA5, Biolegend) and anti-mouse F4/80 antibodies (Clone BM8, Biolegend) at a 1:100 dilution for 30 min on ice. Cells were fixed and permeabilized using Fixation/permeabilization concentrate (Ebioscience) and labeled with anti-Ki67 (Clone SolA15, Ebioscience). The FACS data were acquired using a CantoII flowcytometer (BD) and analyzed with FlowJo software. The living PDGFRα^+^F4/80^−^ CAFs were sorted using Aria II cell sorter (BD Bioscience).

Sorted CAFs were seeded in 24-well plates in DMEM containing 10% FBS and 100 units/ml penicillin/streptomycin. Subsequently, non-adherent cells were removed by extensive washing with PBS and adherent cells were treated as indicated for further analysis.

### Immunofluorescence

For the cell immunofluorescence assay sorted CAFs were seeded in a cell culture dish (NEST) and cultured overnight. The CAFs were fixed in 4% paraformaldehyde for 20 min and incubated with solution (including 1% BSA and 0.3% Triton X-100 in PBS) for permeabilization and blockade of unspecific binding at room temperature for 45 min. The primary antibody against mouse C3aR (14D4, Hycult biotech, 1:50 diluted) and rabbit anti-mouse α smooth muscle actin (E184, Abcam, 1:500 diluted) were incubated at 4 °C overnight. For tumor tissues, frozen sections of mammary tissue from WT/C3aR^−/−^ mice were fixed with ice-cold 4% paraformaldehyde for 15 min at room temperature. After washing with PBS, the sections were blocked with 5% bovine serum albumin-containing PBS for 1 h at room temperature, followed by primary antibody incubation overnight at 4 °C. The following antibodies were used for tissue immunofluorescence staining: E-Cadherin mouse monoclonal antibodies (AF0138, Beyotime, 1:50 diluted) and Vimentin Mouse Monoclonal Antibodies (AF0318, Beyotime, 1:100 diluted). Alexa Fluor 488-labeled goat anti-rabbit IgG (1:200 dilution, Abcam), Alexa Fluor 488-labeled goat anti-Armenian hamster antibody (1:200 dilution, Abcam),or Alexa Fluor647-labeled goat anti-rabbit IgG (1:200 dilution, Abcam) were used as secondary antibodies. After counterstaining with DAPI (Beyotime), sections were imaged under an Olympus Fluorescence Microscope.

### Data availability

RNA-sequencing data has been deposited to NCBI (PRJNA600392).

### Statistical analysis

Data were expressed as the means ± SEM after more than three repeated independent experiments and were analyzed using the GraphPad 7.0 software. Either two-tailed unpaired Students T-tests or other statistical methods indicated were used to evaluate the differences. *P*-values < 0.05 were considered statistically significant.

## Results

### C3aR deficiency reduced metastasis of breast cancer to the lungs

**Fig. 1:**
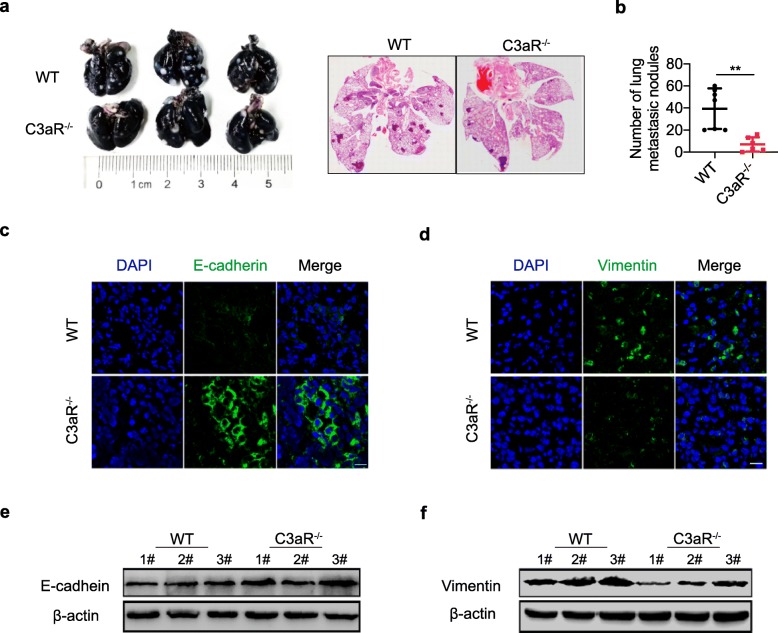
Lung metastasis were reduced in C3aR deficiency breast tumor-bearing mice. **a-f** 4 T1 cells were orthotopically injected into WT (*n* = 7) or C3aR^−/−^ (*n* = 6) mice. Mice were anesthetized and sacrificed 28 days post tumor inoculation. **a** Lung metastases burden in the WT and C3aR^−/−^ tumor-bearing mice, and scans of H&E stained sections of the lungs of WT and C3aR^−/−^ mice with breast tumors. b Quantification of lung metastases. **c-d** Expression of E-Cadherin and Vimentin in the primary tumor tissue detected by immune-fluorescence, and western blot assay (**e-f**). (*P < 0.05, **P < 0.01, ***P < 0.001)

Previously, we and others demonstrate that C3aR signaling promote tumor growth by promoting immune inhibition [6, 10]. However, its role in metastatic spread of breast cancer has not been explored. To investigated whether C3aR signaling contributed to metastasis, we orthotopicaly injected 4 T1 cells (a mouse breast cancer cell line), which closely mimics stage IV of human breast cancer, into mammary fat pad of Balb/c C3aR^+/+^ mice and C3aR^−/−^ mice, respectively. Our result suggest that C3aR deficiency resulted in a decreased lung metastatic burden (Fig. 1a and b), while it did not significantly affect the growth of primary breast tumors (Additional file 1: Figure S1a-d). Epithelial mesenchymal transition(EMT)induction is one of the most important mechanisms for cancer metastasis [22], the downregulation of epithelial marker E-cadherin and the upregulation of mesenchymal marker vimentin are typical characteristics of EMT. To this end, we detected the EMT markers in tumor tissues and found that the mesenchymal marker vimentin was down-regulated, and the epithelial marker E-cadherin was up-regulated when the 4 T1 cell was inoculated in C3aR^−/−^ mice (Fig. 1c-f). The results suggest that C3aR signaling promote metastasis of breast cancer via inducing EMT of tumor cells.

### Involvement of CAFs in C3aR mediated breast cancer lung metastasis

**Fig. 2:**
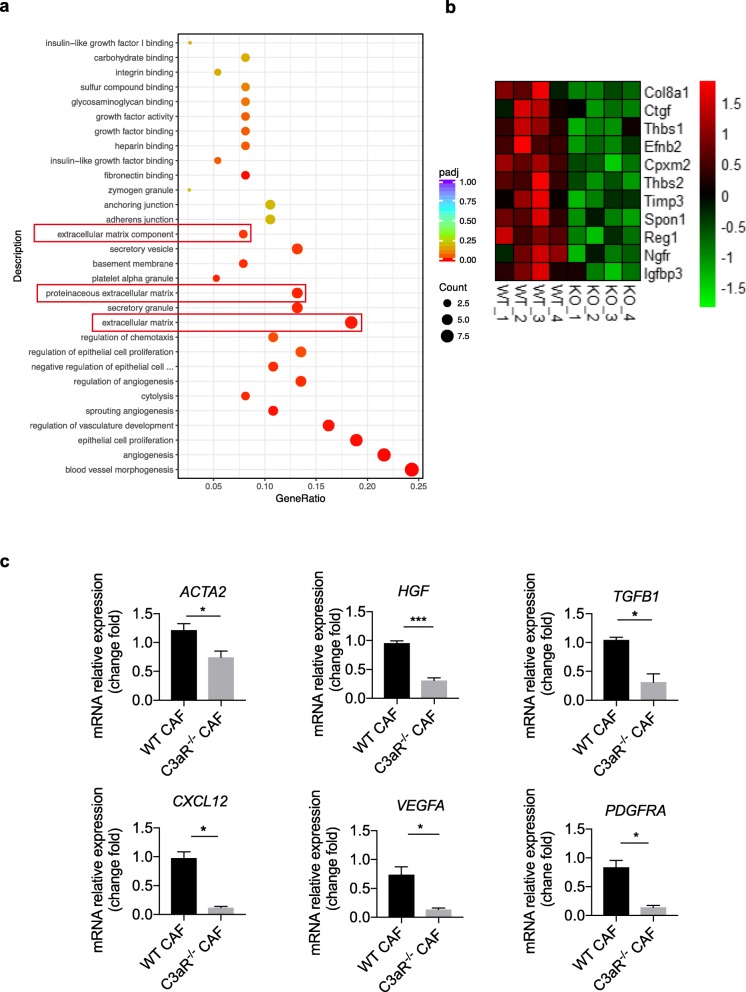
The reduced lung metastasis of breast cancer in C3aR−/− mice is associated with the altered function of CAFs. **a-b** 4 T1 cells were orthotopically injected into WT or C3aR^−/−^ mice. Mice were sacrificed 16 days post tumor inoculation and the tumors were harvested. RNA-sequencing was conducted. **a** Gene ontology enrichment analysis of WT and C3aR^−/−^ 4 T1 tumors. Enrichment scatter plot in which the abscissa is the GeneRatio (the ratio of the number of differential genes on the GO pathway to the total number of differential genes). **b** Heat map of mRNA expression for differential extracellular matrix related genes. **c** qPCR analysis of mRNA levels of CAF markers (*ACTA-2, PDGFRα*) and functional cytokines (*TGFβ, HGF, CXCL12, and VEGF-A*) of CAFs, isolated from WT or C3aR^−/−^ tumors (*P < 0.05,**P < 0.01)

To further illustrate the underling mechanism of C3a-C3aR mediated breast cancer metastasis, we applied RNA sequencing to profile the gene expression of 4 T1 tumors from WT mice or C3aR^−/−^ mice. RNA sequencing data revealed that tumor tissue from C3aR^−/−^ mice displayed significant differences in gene expression profiles compared with that of WT tumors. Among them, we found that genes associated with extracellular matrix components were significantly downregulated in tumors isolated from C3aR^−/−^ mice than those from WT mice **(**Fig. 2a and b). To our knowledge, CAFs is the main source of extracellular matrix components in TME [31]. Hence, we clarified whether C3aR signaling altered the quantity and quality of CAFs within the TME. We firstly compared the percentage of CAFs (PDGFRα^+^F4/80^−^ cells) in tumors isolated from WT and C3aR^−/−^ mice. We found comparable CAFs infiltration between the two groups (Additional file 1: Figure S2a&b). We next determined the mRNA expression of genes, associated with activation and functionality of CAFs, isolated from the 4 T1 tumors from C3aR^−/−^ mice or WT mice (Additional file 1: Figure S2c). Our data indicates that C3aR^−/−^ CAFs expressed a diminished level of conventional fibroblast markers, including a-SMA, PDGFRα, FAP when compared with WT CAFs. Accordingly, C3aR^−/−^ CAFs also expressed reduced functional cytokines including *TGF-β, CXCL12, HGF* (Fig. 2c). These results suggest that C3aR signaling promotes lung metastasis of breast cancer possibly by altering the function of CAF, rather than changing its numbers.

### C3 expression is correlated with CAF activation and function makers in human breast cancer

**Fig. 3.**
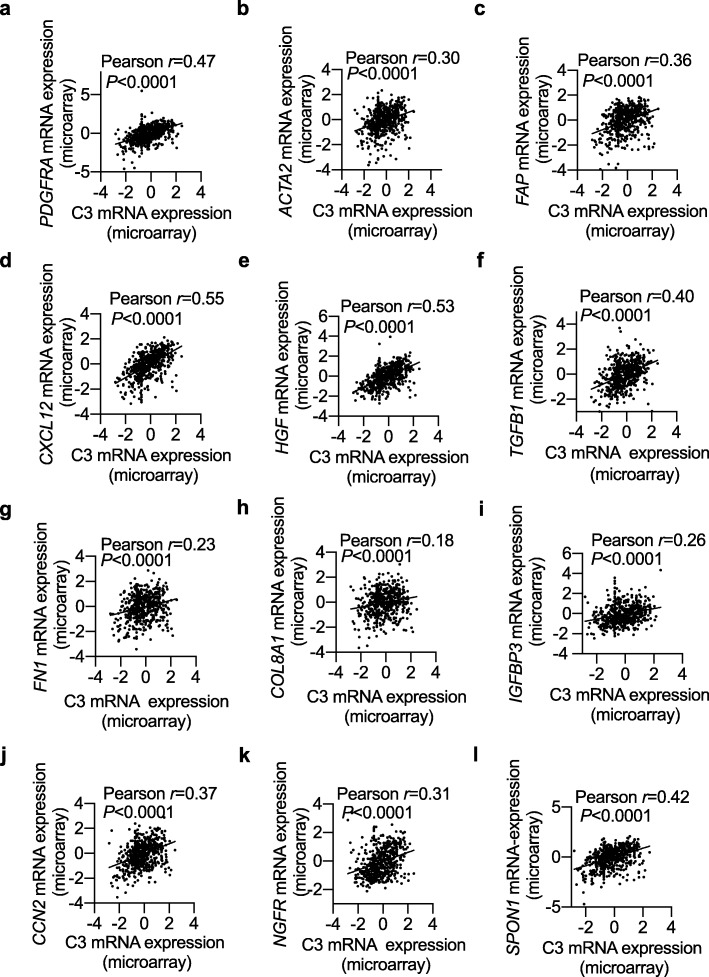
C3 expression is correlated with CAF activation and function in human breast cancer. The relationship between the mRNA transcripts for C3 and phenotypic markers of CAF (**a-c**, *PDGFRA, ACTA2, FAP*), functional cytokines (**d-f**, *TGFB1, CXCL12, HGF,*) and ECM components (**g-l**, *Fn1, Col8a1, IGFBP3, CCN2, NGFR, SPON1*) were determined by Pearson’s correlation analyses. Expression data for these genes in invasive breast cancer patients were obtained from the cBio Cancer Genomics Portal database (*n* = 526). Data were analyzed three times

Upon analysis of the clinical data from human invasive breast cancer mRNA profiles for tumor samples of 526 invasive breast cancer patients, we found that C3 expression was positively correlated with CAFs markers (Fig. 3.)a-c and its effector cytokines(Fig. 3.d-f) in human breast cancer tissues [32]. Additionally, the ECM components (Fig. 3. g-l) mostly synthesized by CAF were also associated with local C3 expression. To sum up, we concluded that production of C3 complement may contribute to enhancing the function of CAF and promoting the formation of invasive breast cancer.

### C3aR signaling is involved in CAFs activation

**Fig. 4:**
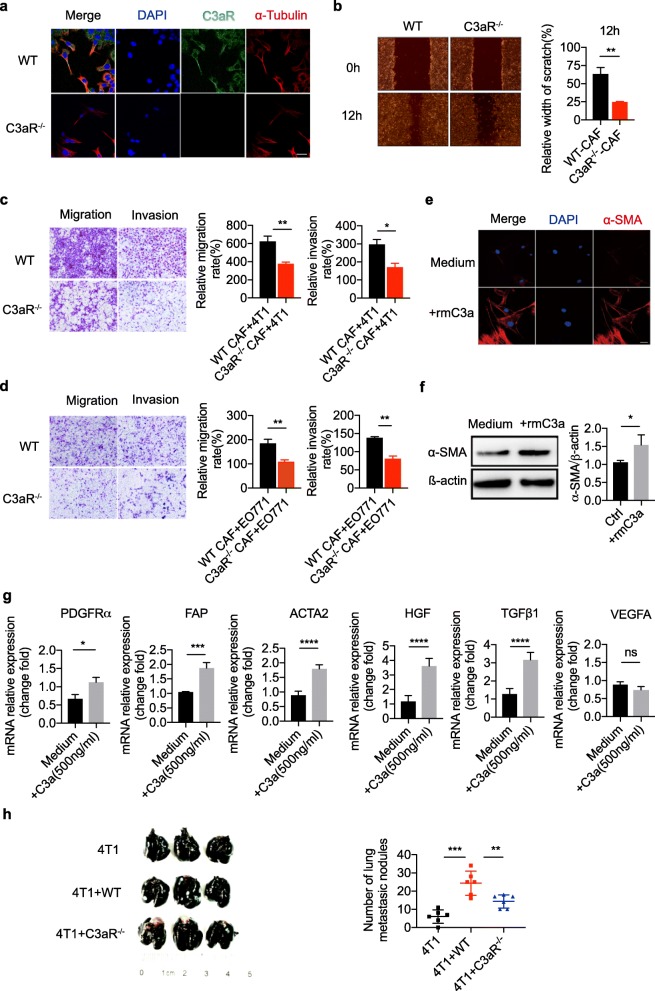
C3aR signaling promotes the pro-meatastatic function of CAF. **a** CAFs were sorted by Flow cytometry as PDGFRa^+^F4/80^−^ cells of 4 T1 tumor tissues from WT or C3aR^−/−^ mice. Immunofluorescence analysis of C3aR expression in WT and C3aR^−/−^ CAF. **b** The migratory properties of 4 T1 cells cultured with WT and C3aR^−/−^ CAFs detected in scratch assays (*P < 0.05). **c-d** The migration and invasive capability of 4 T1/EO771 tumor cells co-cultured with WT CAFs and C3aR^−/−^ CAFs (*P < 0.05). CAF obtained from 4 T1 tumor-bearing WT mice were stimulated with rmC3a(0.5μg/ml) for 24 h, the expression of α-SMA was analyzed by immunofluorescence (**e**) and western blotting assay (**f**). The software ImageJ was used to qualify the signal intensities of the western blot, and the ratio of α-SMA/β-actin is presented. **g** Quantitative PCR analysis of mRNA level of CAF markers (*PDGFRA, FAP, ACTA2*) and functional cytokines (*TGF-β1, HGF, VEGFA*) in treated or untreated CAFs was performed. **h-i** 4 T1 cells were co-injected with CAFs derived from WT or C3aR^−/−^ mice in the mammary fat pad. The number of 4 T1 lung metastasis tumor burden was calculated after 28 days. Data are expressed as the mean ± SEM. (*p < 0.05,**p < 0.01,***p < 0.001)

To illustrate the role of C3aR signaling in modulating CAFs function, we firstly asked whether CAFs express C3aR. To this end, we stained C3aR in sorted PDGFRα^+^ F4/80^−^ cells by immunofluorescence. We found that CAFs cells expressed C3aR, a G-protein coupled receptor, both on the membrane and intracellularly (Fig. 4a). To our knowledge, internalization of C3aR usually suggests that the C3aR receptor is functional as it was reported before [33].

The elevated expression of soluble factors in CAFs has been suggested to act as a possible regulator of adjacent cancer cell migration. To test this, we co-cultured 4 T1 cells or EO771 cells with CAFs isolated from C3aR^+/+^ or C3aR^−/−^ tumor-bearing mice, respectively. Scratch assay and transwell migration/invasion assays confirmed that the co-culturing of C3aR^−/−^ CAFs with 4 T1 cells rarely stimulated breast cancer epithelial cell migration and invasion, while co-culturing of C3aR^+/+^ CAFs significantly promoted EO771 cells migration and invasion (Fig. 4b-d). Moreover, recombinant mouse C3a did not only induce CAFs phenotype markers characterized by alpha smooth muscle actin (a-SMA) expression (Fig. 4e), but it also stimulated the expression and secretion of TGFβ, a crucial pro-metastasis cytokine effector of CAFs. Furthermore, the expression of genes associated with phenotype markers and functional cytokines of CAFs were upregulated under C3a stimulation (Fig. 4g). For the analysis of whether C3aR signaling on CAFs altered the ability of promoting metastasis in-vivo, we injected 4 T1 cells alone, with WT CAFs or with C3aR^−/−^ CAFs orthotopically into the mammary fat pad of immunodeficient nude mice. Co-inoculation of 4 T1 cells with WT CAFs accelerated lung metastasis, compared with 4 T1 alone, while C3aR signaling deficiency in CAFs abrogated its metastasis-promoting effect (Fig. 4h and i). These results indicated that C3a acts directly on CAFs in a C3aR dependent manner.

### PI3K-AKT signaling is involved in C3aR signaling-driven CAFs activation

**Fig. 5:**
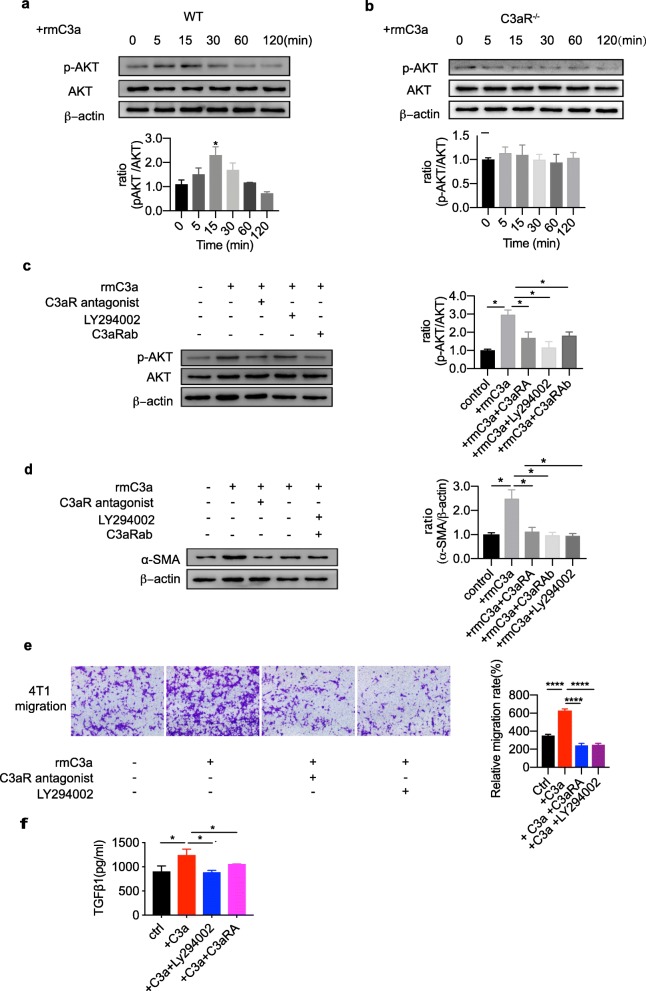
C3aR signaling shifts CAF function via PI3K activation. **a** Dynamic up-regulation of phosphorylated AKT (Ser473) in CAFs stimulated with rmC3a at different time points. **b** Dynamic phosphorylated AKT (Ser473) expression of C3aR^−/−^ CAFs treated with rmC3a at different time points. **c** rmC3a-induced phosphorylated AKT (Ser473) expression of WT CAFs after pretreatment of C3aR antagonist (SB290157), PI3K inhibitor (LY294002), or C3aR antibodies (14D4). **d** α-SMA expression of C3a-induced CAFs blocked with C3aR antagonist, PI3K inhibitor, or C3aR antibodies. (*p < 0.05) (**e**) Transwell assay demonstrated that rmC3a treated CAF facilitate migration capacity of 4 T1 cells, and can be inhibited by C3aR antagonist and PI3K inhibitor. (**p < 0.01,***p < 0.001,****p < 0.0001) (**f**)TGF-ß1 secretion of CAF stimulated by rmC3a after blockade with C3aR antagonist or PI3K inhibitor (LY294002), compared with medium only. (*p < 0.05)

We found that CAFs were stimulated with rmC3a protein and showed an increased phosphorylation of AKT at Ser473 as early as 5 min, reaching a peak at 15 min (Fig. 5a). However, rmC3a stimulation could not activate PI3K signaling in C3aR^−/−^ CAFs (Fig. 5b). Pretreatment with PI3K inhibitor (LY294002) inhibited increased expression of α-SMA and TGFβ secretion induced by C3a. To further determine whether C3a-mediated AKT phosphorylation is C3aR-dependent, we used C3aR antibody (14D4) or a C3aR antagonist (SB290157) to block C3aR signaling. Our data suggest that both C3aR antagonist and C3aR antibody pretreatment could inhibit C3a-mediated AKT phosphorylation (Fig. 5c). Similarly, C3a-induced α-SMA and TGFβ1 were greatly suppressed after C3aR blockade or PI3K inhibition (Fig. 5d and f). C3a stimulated CAF facilitated migration capacity of 4 T1 cells, and can be inhibited by C3aR antagonist and PI3K inhibitor. These data suggest C3a plays a crucial role in regulating CAF activation and effector cytokine production via activating PI3K/AKT signaling pathway.

### Pharmacological inhibition of C3aR signaling inhibits breast cancer metastasis

**Fig. 6:**
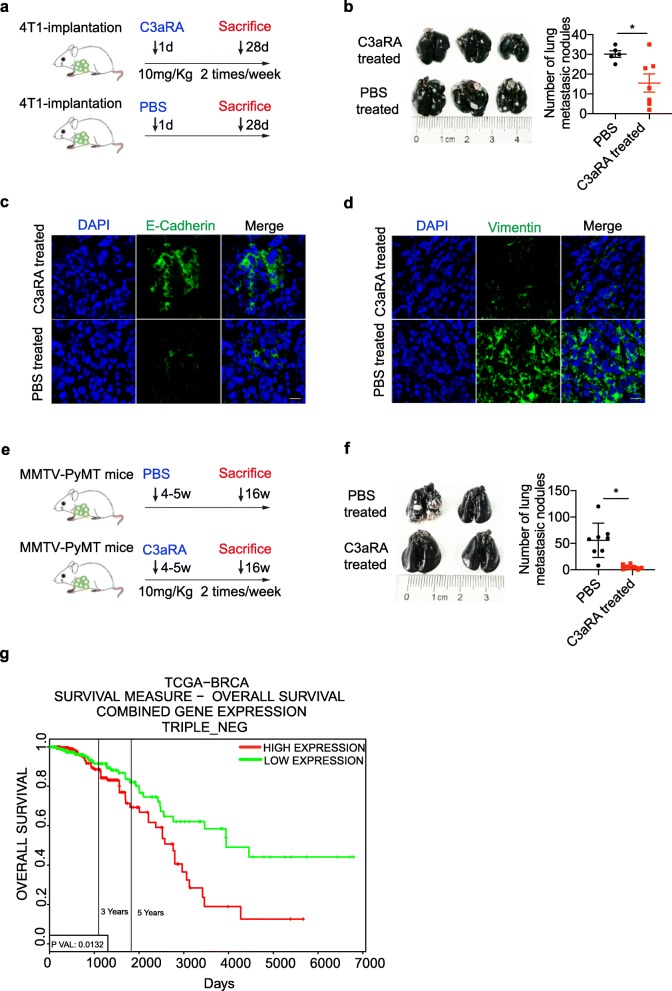
Targeting C3aR Inhibits Lung Metastasis of Breast Cancer. **a** 4 T1 cells were inoculated into the third mammary fat pad of mice on day 0. C3aRA or PBS were injected i.p.10 mg/kg bodyweight from day 1, twice a week. **b** 28 days after tumor inoculation, lung metastasis burden in the C3aRA treated (*n* = 7) or PBS injected (*n* = 6) tumor-bearing mice. **c-d** Vimentin and E-Cadherin expression were detected by immunofluorescence assay. Data shown as three repeated experiments. **e** 4–5-week-old female MMTV-PyMT mice were intraperitoneally injected with (10 mg/Kg body weight) C3aR antagonist twice a week. **f** The experimental mice were euthanized at 16 weeks of age, the lungs were inflated with India ink, and the white nodules visible in the lungs represented the metastases burden. (*P < 0.05). **g** Kaplan–Meier curve for survival of human triple negative breast cancer patients from the TCGA-BRAC data sets. Data were obtained from the PROGgeneV2 web application and C3aR1 and PDGFA gene co-expression was divided into high and low at the median expression. n represents the number of patients at day 0. *P*-values less than 0.05 were considered significant

To address the possible utility of targeting C3aR as a translational anti-metastasis strategy, we employed a C3aR antagonist (SB290157), used in studies of reactive airway [34] and leptomeningeal metastasis [12], to treat a 4 T1 orthotopic mouse model (Fig. 6a). Our data suggest that lung metastasis was markedly reduced by treatment with C3aR antagonist (Fig. 6b-d), whereas the difference in the tumor weight was not significantly changed (Additional file 1: Figure S3a). In addition, C3aR inhibition also protected against the development of lung metastasis in the PyMT-MMTV mouse spontaneous breast cancer model (Fig. 6e and f). Additionally, we explored TCGA data and analyzed the correlation between C3aR1^+^PDGFA^+^ expression and the survival of triple negative breast cancer patients, we found that patients with a high level of C3aR1^+^PDGFA^+^ expression had a poorer survival rate **(**Fig. 6g). These results potentiate C3aR signaling blockade as an effective anti-metastasis strategy in breast cancer management.

## Discussion

Breast cancer related deaths are primarily attributed to metastasis [35]. It is well known that CAFs form the major population of tumor stromal cells and serve as a main source of tumor extracellular matrix components [31, 36]. Crosstalk between cancer cells and CAFs is involved in the metastasis of breast cancer [37], yet the master regulators and their underlying mechanisms remain largely unknown. In this study, we demonstrated that C3a-C3aR signaling facilitates the metastasis of breast cancer via modulating CAFs function. Activation of PI3K/AKT signaling was involved in C3a-C3aR signaling which drive CAF activation. Moreover, previous studies demonstrated that PI3K-AKT signaling is involved in CAFs’ function [10, 30], and we and others have revealed that C3aR signaling could promote PI3K-AKT activation [9]. Genetic or pharmacological blockade of C3aR signaling effectively inhibited lung metastasis in breast cancer mouse models. Our findings suggest C3aR signaling might be a promising anti-metastatic strategy in breast cancer therapy.

Previously, complement activation has been identified as an important element in infection [1, 38]. However, new insights regarding the mechanism of complement activation have revealed an additional complex level involved in biology and its impact on diseases. A series of complement components accumulated in TME determined tumor development by binding to their corresponding receptors. Our findings were in line with those from previous studies which demonstrated that C3a-C3aR signaling promotes tumor growth via the alteration of TME [6, 7, 9, 33, 39]. It was reported that B16 tumor growth in C3aR deficient mice was suppressed by relieving the neutrophil and CD4^+^ T cell responses [14]. Additionally, C3aR blockade delayed tumor growth of orthotopic inoculated CMT and LLC in an immunocompetent model of lung cancer [7]. Finally, intracellular activation of complement C3 contributed to tumor growth via the modulation of tumor associated macrophages [10, 40] . A recent study demonstrated that tumor-cell-derived C3 activates the C3a receptor of the choroid plexus epithelium to disrupt the blood-CSF barrier, which promotes leptomeningeal metastasis [12]. However, whether and how C3a-C3aR signaling promotes the metastasis of breast cancer remains unknown.

In this study, we demonstrated that C3a-C3aR signaling promotes breast cancer metastasis via the modulation of CAFs. CAFs are the largest population of stromal cells within breast cancers and accumulating evidence suggest that CAFs play a critical role in cancer metastasis by releasing cytokines, chemokines, and extracellular matrix [41]. Our findings suggest that C3a-C3aR signaling promotes the activation of CAFs as characterized by enhanced expression of activation markers, such as alpha-SMA. Consistent with our results, previous studies have revealed a key role of C3a-C3aR signaling in the promotion of fibrosis, a process orchestrated by activated fibroblast.

Accumulating evidence suggest that EMT induction is one of the most important mechanisms for cancer metastasis [22, 42], and the down-regulation of epithelial marker E-cadherin and the upregulation of mesenchymal marker vimentin are typical characteristics of EMT [43]. Our data suggest that C3aR signaling activated CAFs augmenting metastasis by promoting EMT of tumor cells. EMT is one of the major mechanisms in tumor metastasis, and TGFβ has been approved as a critical promoter for transforming epithelial cells into mesenchymal cells [24]. We assumed that C3aR signaling activated CAFs facilitated EMT of tumor cells via TGFβ, which was induced by recombinant mouse C3a in-vitro. Decreased secretion of pro-metastasis factors (such as TGF-β, platelet-derived growth factor [PDGF], hepatocyte growth factor [HGF]) involved in CAFs activity were observed in the C3aR deficiency CAFs in the 4 T1-bearing model. Moreover, increasing TGF-β and CAF markers were found in C3a-treated CAFs in vitro. Importantly, the effects of C3aR signaling on metastasis, independent from mechanisms operating in primary tumors, did not delay the growth of the primary tumor. This finding, which is in contrast with our previously reported findings, can be attributed to the difference in tumor type, as was previously reported for C5aR signaling in cancer [44].

Mechanically, we identified that PI3K/AKT signaling plays an essential role in C3a/C3aR signaling mediated the activation of CAFs. Our data suggest that recombinant C3a induced the phosphorylation of AKT and C3aR antibodies. Moreover, increasing TGF-β and CAF markers were found in C3a-treated CAFs in vitro. Additionally, C3a-stimulated elevation of TGFβ and migration capacity of 4 T1 cells can be blocked by C3aR antibodies and C3aR antagonists assist with the suppression of AKT-phosphorylation. Consistent with our data, previous reports showed that alterations in the PI3K/AKT pathway are involved in the activation of tumor stromal cells [45].

### Conclusion

In summary, our study showed that C3aR signaling plays a unique role in promoting lung metastasis of breast cancer by modulating CAFs. C3aR deficiency inhibits pro-metastatic cytokines production by CAFs in a 4 T1 tumor model. Moreover, increased expression of TGF-β and CAF markers were found in C3a-treated CAFs in vitro. Mechanically, we identified that PI3K/AKT signaling plays an essential role in C3a-C3aR signaling mediated by CAFs activation. Our data demonstrated that targeting C3aR might be an effective strategy in tumor metastasis control in breast cancer.

## Additional file

**Additional file 1: Table S1.** Listing of primers used in this study. **Figure S1.** Breast cancer development in C3aR deficient mice. **a** 4 T1 cells were orthotopically injected into WT or C3aR^−/−^ mice. Tumor volumes of WT and C3aR^−/−^ mice were monitored at various time points after tumor inoculation. **b** On day 28 post tumor challenge, the tumor size and weight of these mice were investigated. **c** Tumors were harvested on day 15 after 4 T1 cell inoculation and single cell suspension was prepared for flow cytometry staining. The gating strategy for living cells is shown. **d** Percentage of Ki67^+^ cells in CD45^−^ tumor cells in WT (*n* = 8) and C3aR^−/−^ (*n* = 7) mice detected by FACs. **Figure S2.** The proliferation of CAF cells in C3aR^−/−^ mice was comparable with that of WT mice. **a** Tumors were harvested on day 15 after 4 T1 cell inoculation and single cell suspension was prepared for flow cytometry staining. CAF was defined as PDGFRa^+^F4/80^−^. **b** Percentage of PDGFRα^+^F4/80^−^ in total living cells from C3aR^−/−^ and WT tumor-bearing mice. **c** Tumors were harvested on day 15 after 4 T1 cell inoculation and CAFs were sorted from FACS. The purity of sorted CAF was shown. **Figure S3.** C3aRA treatment has no effect on the breast cancer growth in 4 T1-bearing mice. **a** Tumor growth of 4 T1-bearing mice in C3aRA treated or PBS treated group. **b** Tumor weight of the two group of mice.

## Data Availability

The datasets used and/or analyzed during the current study are available from the corresponding author on reasonable request.

## Abbreviations

C3a: Complement 3a
C3aRA: Compelment 3 receptor antagonist
CAF: Carcinoma associated fibroblast
EMT: Epithelial-Mesenchymal Transition
HGF: Hepatocyte growth factor
TGF: Transforming growth factor
TME: Tumor microenviroment
