# Supplementary material for: C3a-C3aR signaling promotes breast cancer lung metastasis via modulating carcinoma associated fibroblasts
Source: J Exp Clin Cancer Res. 2020 Jan 13;39:11. doi: 10.1186/s13046-019-1515-2 (PMC6958674; doi:10.1186/s13046-019-1515-2)
Supplement: Supplementary file 1 — Additional file 1: Table S1. Listing of primers used in this study. Figure S1. Breast cancer development in C3aR deficient mice. a 4 T1 cells were orthotopically injected into WT or C3aR−/− mice. Tumor volumes of WT and C3aR−/− mice were monitored at various time points after tumor inoculation. b On day 28 post tumor challenge, the tumor size and weight of these mice were investigated. c Tumors were harvested on day 15 after 4 T1 cell inoculation and single cell suspension was prepared for flow cytometry staining. The gating strategy for living cells is shown. d Percentage of Ki67+ cells in CD45− tumor cells in WT (n = 8) and C3aR−/− (n = 7) mice detected by FACs. Figure S2. The proliferation of CAF cells in C3aR−/− mice was comparable with that of WT mice. a Tumors were harvested on day 15 after 4 T1 cell inoculation and single cell suspension was prepared for flow cytometry staining. CAF was defined as PDGFRa+F4/80−. b Percentage of PDGFRα+F4/80− in total living cells from C3aR−/− and WT tumor-bearing mice. c Tumors were harvested on day 15 after 4 T1 cell inoculation and CAFs were sorted from FACS. The purity of sorted CAF was shown. Figure S3. C3aRA treatment has no effect on the breast cancer growth in 4 T1-bearing mice. a Tumor growth of 4 T1-bearing mice in C3aRA treated or PBS treated group. b Tumor weight of the two group of mice. [file 13046_2019_1515_MOESM1_ESM.docx]

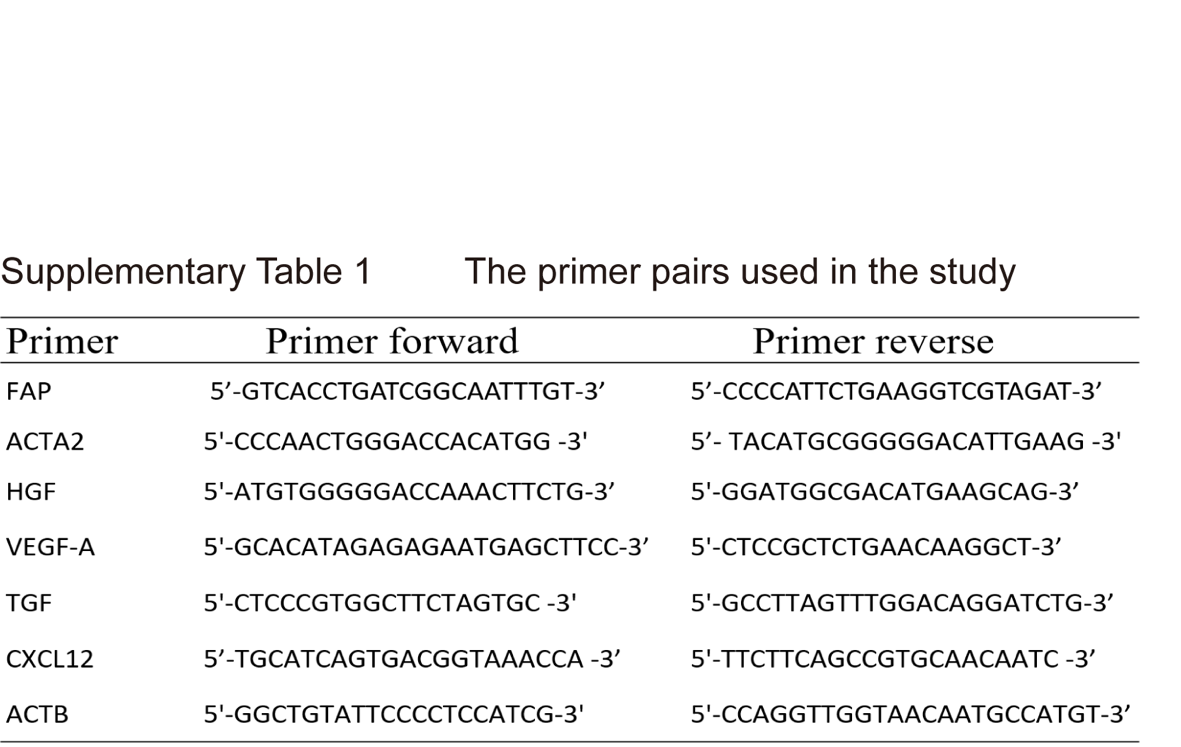


Supplementary table 1. Listing of primers used in this study


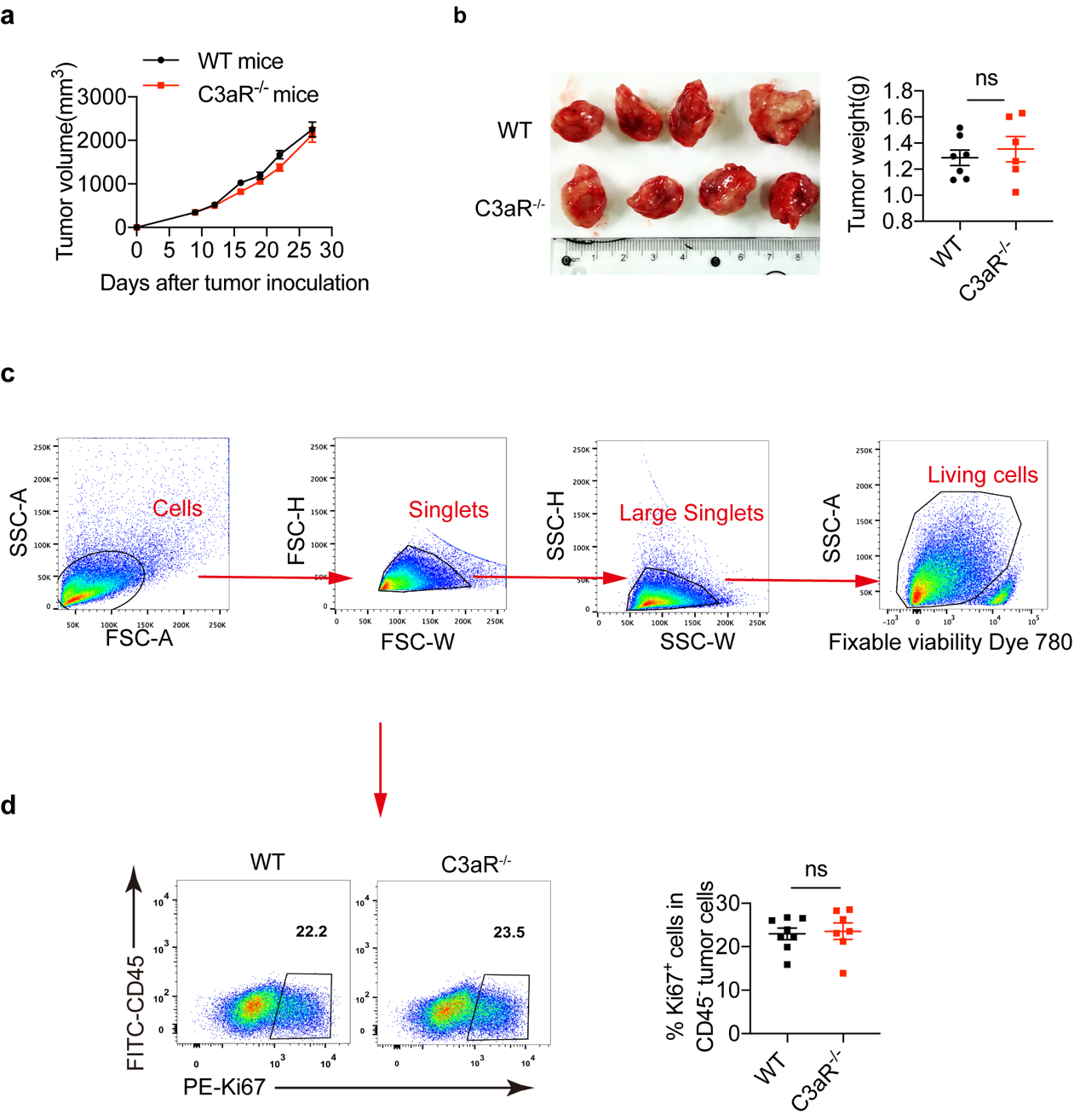
 **Figure S1.** **Breast cancer development in C3aR deficient mice. a** 4T1 cells were orthotopically injected into WT or C3aR^-/-^ mice. Tumor volumes of WT and C3aR^-/-^ mice were monitored at various time points after tumor inoculation. **b** On day 28 post tumor challenge, the tumor size and weight of these mice were investigated. **c** Tumors were harvested on day 15 after 4T1 cell inoculation and single cell suspension was prepared for flow cytometry staining. The gating strategy for living cells is shown. **d** Percentage of Ki67^+^ cells in CD45^-^ tumor cells in WT (n=8) and C3aR^-/-^ (n=7) mice detected by FACs.


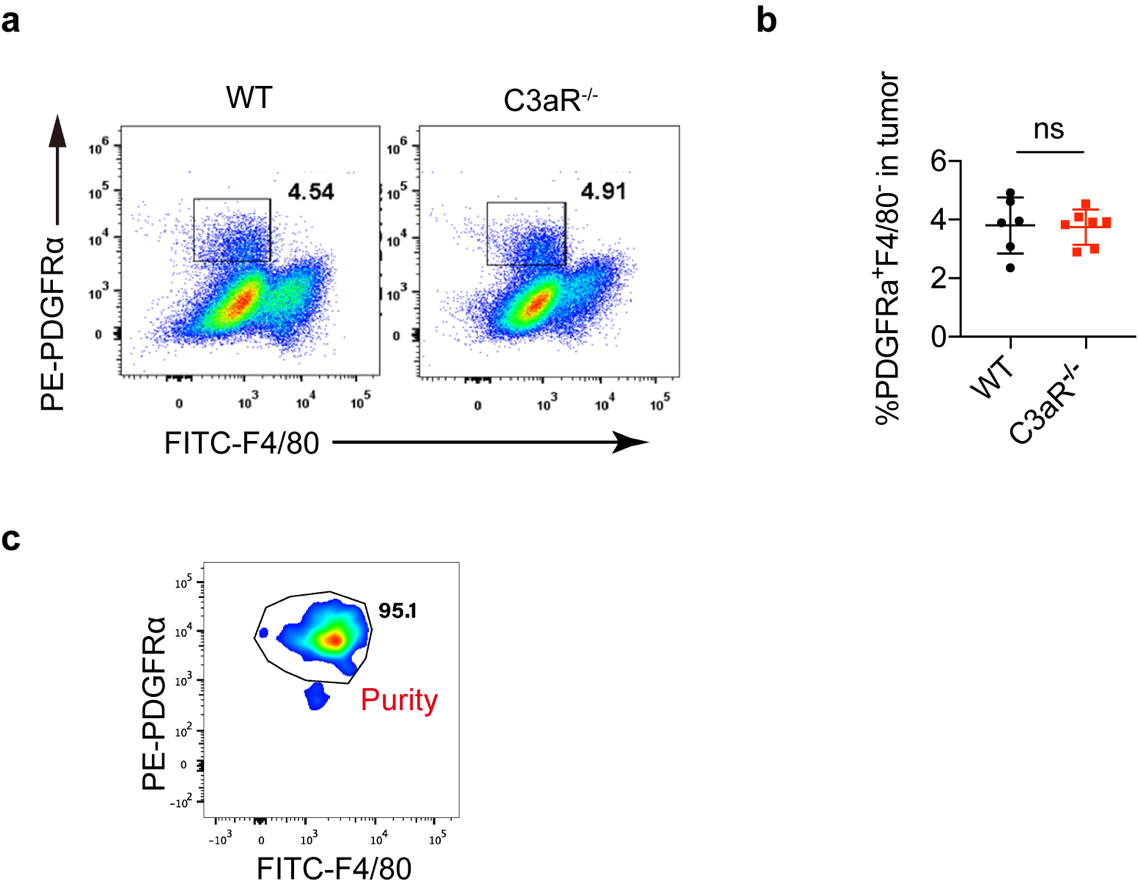


**Figure S2.** **The proliferation of CAF cells in C3aR^-/-^ mice was comparable with that of WT mice. a** Tumors were harvested on day 15 after 4T1 cell inoculation and single cell suspension was prepared for flow cytometry staining. CAF was defined as PDGFRa^+^F4/80^-^. **b** Percentage of PDGFRα^+^F4/80^-^ in total living cells from C3aR^-/-^ and WT tumor-bearing mice. **c** Tumors were harvested on day 15 after 4T1 cell inoculation and CAFs were sorted from FACS. The purity of sorted CAF was shown.


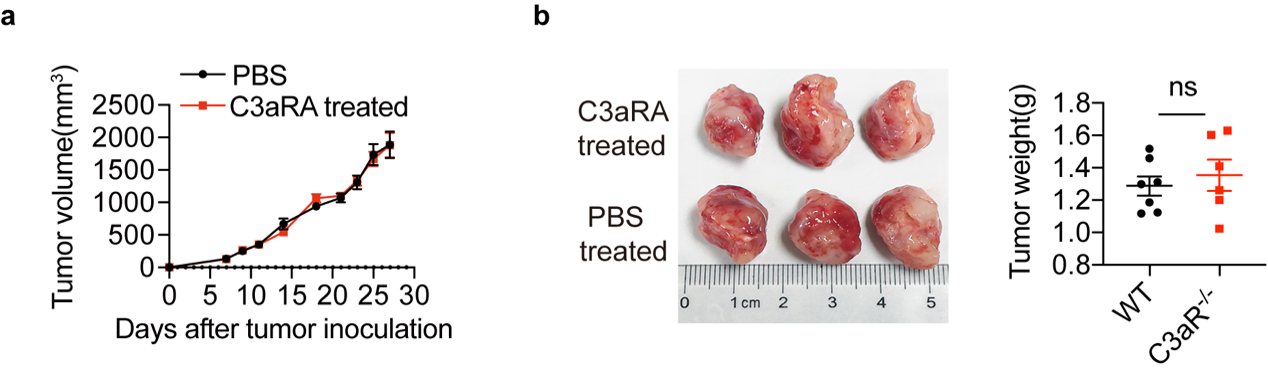


**Figure S3.** **C3aRA treatment has no effect on the breast cancer growth in 4T1-bearing mice. a** Tumor growth of 4T1-bearing mice in C3aRA treated or PBS treated group. **b** Tumor weight of the two group of mice.
